# Supplementary material for: Liquid-Liquid Phase Separation of Patchy Particles Illuminates Diverse Effects of Regulatory Components on Protein Droplet Formation
Source: Sci Rep. 2018 Apr 30;8:6728. doi: 10.1038/s41598-018-25132-1 (PMC5928213; doi:10.1038/s41598-018-25132-1)
Supplement: Supplementary file 1 — Supplementary Information [file 41598_2018_25132_MOESM1_ESM.pdf]

Liquid-Liquid Phase Separation of Patchy Particles Illuminates Diverse Effects of Regulatory Components on Protein Droplet Formation

Valery Nguemaha[1] and Huan-Xiang Zhou[1,2,*]

[1]Department of Physics and Institute of Molecular Biophysics, Florida State University, Tallahassee, FL 32306, United State

[2]Department of Chemistry and Department of Physics, University of Illinois at Chicago, Chicago, IL 60607, United States

Supplementary Information

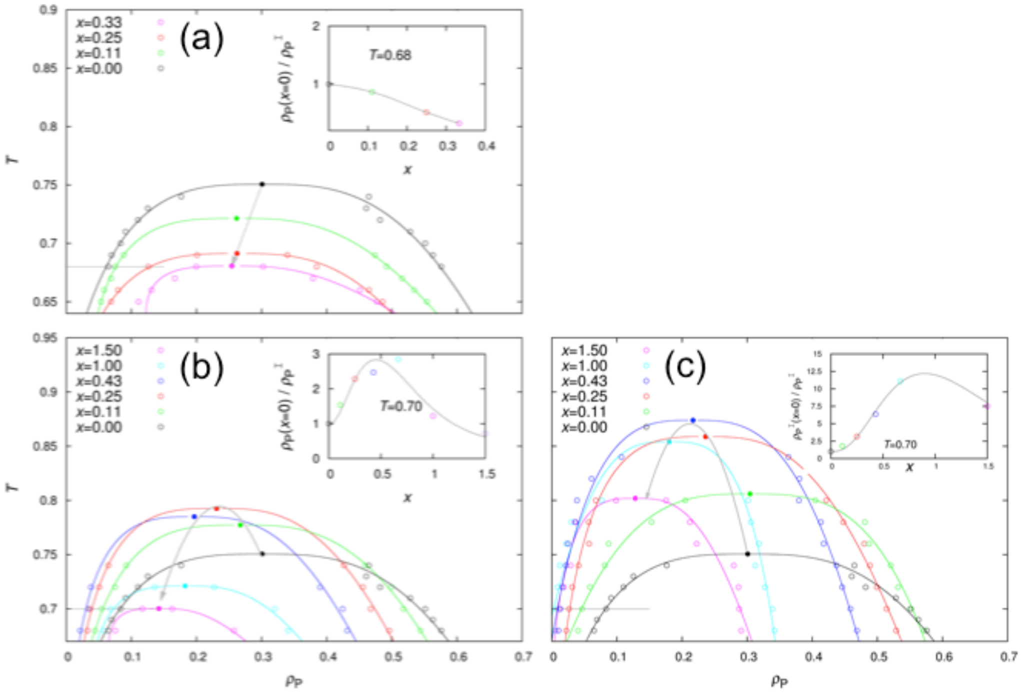

Supplementary Figure S1. Phase diagrams. Results at ε_{PR} = 0.7, 1.25, and 1.5 are shown in **(a)** – **(c)**, respectively. Open symbols are calculation results, and solid curves are fits to equation (7). The critical points from the fits are shown as filled symbols, with an arrowed gray line or parabola indicating the change with increasing *X*. A horizontal line through the left arm of the binodal indicates the temperature chosen for displaying results in the inset. Inset: ratio of threshold P concentrations between the pure P system and P-R mixture; a smooth curve is drawn to guide the eye.
